# *Notes from the Field:* Severe Health Outcomes Linked to Consumption of Mushroom-Based Psychoactive Microdosing Products — Arizona, June–October 2024

**DOI:** 10.15585/mmwr.mm7401a3

**Published:** 2025-01-09

**Authors:** Heather L. Walker, Maureen Roland, Steven Dudley, Kenneth Komatsu, Joli Weiss, Jamaica Dillard, Hsin-I Lin, Laura Rust, Traci Plummer, Rachel Berg, Stephen Everett, Arthur Chang, Michael Yeh, Johnni Daniel, Shane Brady

**Affiliations:** ^1^Arizona Department of Health Services; ^2^Epidemic Intelligence Service, CDC; ^3^Maricopa County Department of Public Health, Phoenix, Arizona; ^4^Banner Poison and Drug Information Center, Phoenix, Arizona; ^5^Arizona Poison and Drug Information Center, Tucson, Arizona; ^6^Yavapai County Community Health Services, Prescott, Arizona; ^7^Division of Environmental Health Science and Practice, National Center for Environmental Health, CDC.

**Summary**

**What is already known about this topic?**

Availability of products containing labeled and sometimes unlabeled psychoactive compounds is increasing.

**What is added by this report?**

In June 2024, Arizona identified a cluster of cases of severe adverse health effects, including neurologic and cardiac signs and symptoms, after ingestion of Diamond Shruumz–brand chocolate bars. These products are labeled to include psychoactive mushroom extracts. The investigation prompted a nationwide product recall and public health response with detection of 180 cases in 34 states.

**What are the implications for public health practice?**

Edible products marketed as containing mushroom-based psychoactive substances could provoke life-threatening illness. Persons should stop consuming Diamond Shruumz–brand products and exercise caution when consuming other products reported to contain mushroom-based psychoactive substances.

In June 2024, Arizona Department of Health Services (ADHS) was notified by Arizona’s poison control centers (PCCs)* of adverse health outcomes occurring after ingestion of a Diamond Shruumz–brand product containing proprietary blends of mushroom extracts and adaptogens.^†^ These products were sold as chocolate bars, gummies, and cones and could be purchased online or at local retailers nationwide.^§^ Availability of similar products containing psychoactive compounds is increasing, with some known to contain unlabeled psychoactive substances (*1*,*2*). This report describes findings from a national outbreak of illness associated with ingestion of Diamond Shruumz–brand products. This activity was reviewed by CDC, deemed not research, and was conducted consistent with applicable federal law and CDC policy.^¶^

## Investigation and Outcomes

On June 1, 2024, a woman (patient A) and a man (patient B), both aged 20–29 years from Yavapai County, Arizona, reported sharing a Diamond Shruumz–brand chocolate bar** (Table). Patient A experienced loss of consciousness and bladder and bowel incontinence and was transported to a local emergency department (ED) by patient B. After arrival at the ED, both patients experienced generalized seizures. Both received benzodiazepines for seizure control and supportive care. Patient A stabilized 10 hours later and was discharged, whereas patient B remained unresponsive, requiring endotracheal intubation and admission to an intensive care unit. Patient B regained consciousness after 8 hours and was discharged after 2 days.

**TABLE:** Characteristics, symptomology, and product consumption results among four patients who consumed Diamond Shruumz–brand products — Arizona, June 2024

Characteristic	Patient A	Patient B	Patient C	Patient D
**Age group**	Young adult	Young adult	Adolescent	Adolescent
**Sex**	Female	Male	Female	Female
**County of residence**	Yavapai	Yavapai	Yavapai	Yavapai
**Diamond Shruumz–brand product consumed/Flavor**	Chocolate bar/Cookies and cream	Chocolate bar/Cookies and cream	Chocolate bar/Dark chocolate	Chocolate bar/Dark chocolate
**Amount consumed**	Eight pieces	Seven pieces	15 pieces	15 pieces
**Symptom onset date**	Jun 1, 2024	Jun 1, 2024	Jun 2, 2024	Jun 2, 2024
**Signs and symptoms associated with product consumption**	Bladder and bowel incontinence, LOC, seizure, and hypersalivation	LOC, seizure, hypersalivation, bradycardia, myoclonus, and diaphoresis	Hallucinations, nausea, vomiting, seizure, muscle rigidity, tachycardia, HTN, agitation, and myoclonus	Hallucinations, nausea, vomiting, seizure, muscle rigidity, tachycardia, HTN, agitation, and myoclonus
**Hospitalized**	Yes	Yes	Yes	Yes
**No. of days hospitalized**	1	2	2	2
**Endotracheal intubation**	No	Yes	Yes	Yes
**Reported substance use history**	Other edible microdosing products	Other edible microdosing products and marijuana	Unknown	Marijuana

**Abbreviations: **HTN = hypertension; LOC = loss of consciousness.

On June 2, 2024, two adolescent girls (patients C and D) from Yavapai County reported ingesting a Diamond Shruumz–brand chocolate bar. Within 2 hours, both experienced decreased level of consciousness, respiratory depression, and vomiting. Patient C also had a generalized seizure. Emergency medical services were called, and both patients were transported to a local ED. En route, patient D had a generalized seizure, and both patients were administered benzodiazepines during transport. In the ED, both patients had tachycardia, hypertension, and intermittent muscle rigidity lasting for 12 hours after ingestion. Both received endotracheal intubation for airway protection, additional benzodiazepine doses for seizure control and muscle rigidity, and supportive care. Both stabilized 24 hours after ingestion and were discharged the next morning.

## Preliminary Conclusions and Actions

On June 3, 2024, PCCs notified ADHS of these patients, issued a press release warning consumers about Diamond Shruumz–brand products, and encouraged health care professionals to report related cases to PCCs (*3*). On June 19, ADHS released a consumer alert including similar messaging (*4*). Two additional patients who sought medical care after consumption of a Diamond Shruumz–brand product were reported to PCCs, one of whom consumed the product and received medical treatment 2 months before this investigation began. ADHS collaborated with PCCs to interview patients, family members, and attending clinicians to collect information on demographic characteristics, medical history, product consumption, patient signs and symptoms, and substance use history. ADHS used syndromic surveillance to identify additional cases not reported to PCCs.

The successful partnership between ADHS and Arizona’s PCCs (*5*) led to prompt notification of this outbreak, highlighting the benefits of collaboration and cooperation with PCCs for investigating poisonings, toxic substance exposures, or ingestions. In addition, PCCs provided lifesaving medical management recommendations to the treating physicians. ADHS alerted CDC and the Food and Drug Administration (FDA) of the ill patients and product consumption, which resulted in a federally led outbreak response that included CDC, FDA, state and local health departments, and regional poison centers. CDC’s National Center for Environmental Health supported the response nationally through creation of a case definition and use of the National Poison Data System for case ascertainment and reporting.^††^ On June 7, 2024, FDA reported on the investigation of illnesses and recommended that consumers not eat, sell, or serve the implicated products.^§§^^,^^¶¶^ On June 12, CDC informed clinicians and public health professionals about this investigation associated with Diamond Shruumz–brand products via the Health Alert Network.*** On June 27, Prophet Premium Blends issued a national recall and ceased production and distribution of all Diamond Shruumz–brand products.^†††^ As of October 31, CDC had identified 180 cases and three potentially associated deaths in 34 states related to the consumption of Diamond Shruumz–brand products.^§§§^ It is important that persons stop consuming Diamond Shruumz–brand products and exercise caution when consuming other products marketed with mushroom-based psychoactive substances.

## Footnotes

* Arizona Poison and Drug Information Center and Banner Poison and Drug Information Center are the two poison centers in Arizona.

†
https://my.clevelandclinic.org/health/drugs/22361-adaptogens

§ Local retailers include those that sell hemp-derived products (cannabidiol or delta-8 tetrahydrocannabinol) and smoke and vape products. Additional information about Diamond Shruumz–brand products is not available because the company’s website is not active at the time of this report.

¶ 45 C.F.R. part 46, 21 C.F.R. part 56; 42 U.S.C. Sect. 241(d); 5 U.S.C. Sect. 552a; 44 U.S.C. Sect. 3501 et seq.

** An entire chocolate bar consists of 15 presectioned pieces with a total weight of 1.6 oz (43.4 g), although some other Diamond Shruumz chocolate bars were divided into 12 presectioned pieces. The company’s website mentioned two squares as the starting dose for “microdosing” and to consume more as needed to achieve the desired effect, with no maximum dose listed.

††
https://www.cdc.gov/chemical-radiological-surveillance/php/about/index.html

§§
https://www.fda.gov/food/outbreaks-foodborne-illness/investigation-illnesses-diamond-shruumz-brand-chocolate-bars-cones-gummies-june-2024

¶¶ https://www.fda.gov/food/hfp-constituent-updates/fda-alerts-industry-and-consumers-about-use-amanita-muscaria-or-its-constituents-food

***
https://emergency.cdc.gov/han/2024/han00509.asp

†††
https://www.fda.gov/safety/recalls-market-withdrawals-safety-alerts/prophet-premium-blends-recalls-diamond-shruumz-products-because-possible-health-risk

§§§
https://www.cdc.gov/environmental-health-studies/outbreak-investigation-diamond-shruumz-products/index.html
